# An Improved YOLOv8-Based Method for Detecting Pests and Diseases on Cucumber Leaves in Natural Backgrounds

**DOI:** 10.3390/s25051551

**Published:** 2025-03-02

**Authors:** Jiacong Xie, Xingliu Xie, Wu Xie, Qianxin Xie

**Affiliations:** 1 Guangxi Key Laboratory of Trusted Software, Guilin University of Electronic Technology, Guilin 541004, China; 1422591832@mails.guet.edu.cn (J.X.); 22032303114@mails.guet.edu.cn (X.X.); wxie@guet.edu.cn (W.X.); 2 School of Computer Science and Information Security, Guilin University of Electronic Technology, Guilin 541004, China

**Keywords:** cucumber leaves, pests and diseases, YOLOv8, deformable convolution, attention mechanism, loss function

## Abstract

The accurate detection and identification of pests and diseases on cucumber leaves is a prerequisite for scientifically controlling such issues. To address the limited detection accuracy of existing models in complex and diverse natural backgrounds, this study proposes an improved deep learning network model based on YOLOv8, named SEDCN-YOLOv8. First, the deformable convolution network DCNv2 (Deformable Convolution Network version 2) is introduced, replacing the original C2f module with an improved C2f_DCNv2 module in the backbone feature extraction network’s final C2f block. This enhances the model’s ability to recognize multi-scale, deformable leaf shapes and disease characteristics. Second, a Separated and Enhancement Attention Module (SEAM) is integrated to construct an improved detection head, Detect_SEAM, which strengthens the learning of critical features in pest and disease channels. This module also captures the relationship between occluded and non-occluded leaves, thereby improving the recognition of diseased leaves that are partially obscured. Finally, the original CIOU loss function of YOLOv8 is replaced with the Focaler-SIOU loss function. The experimental results demonstrate that the SEDCN-YOLOv8 network achieves a mean average precision (mAP) of 75.1% for mAP50 and 53.1% for mAP50-95 on a cucumber pest and disease dataset, representing improvements of 1.8 and 1.5 percentage points, respectively, over the original YOLOv8 model. The new model exhibits superior detection accuracy and generalization capabilities, with a model size of 6 MB and a detection speed of 400 frames per second, fully meeting the requirements for industrial deployment and real-time detection. Therefore, the SEDCN-YOLOv8 network model demonstrates broad applicability and can be effectively used in large-scale real-world scenarios for cucumber leaf pest and disease detection.

## 1. Introduction

The cucumber (*Cucumis sativus*) is one of the most important crops in China and worldwide, valued for its high nutritional, medicinal, and ornamental purposes. The cultivation history of the cucumber can be traced back over 2000 years to the Han Dynasty, when Zhang Qian brought cucumber seeds back from the western regions [1,2]. Due to its diverse applications, cucumber is widely cultivated across China, which is both a major producer and exporter of the crop. In 2020, China had a cucumber cultivation area of 1.2802 million hectares, yielding 72.833 million tons [3]. In 2021, cucumber exports reached 44,649.21 tons. With such high production and significant economic benefits, the effective management of the pests and diseases affecting cucumber leaves has become increasingly important. Common pests and diseases include the target spot [4], leaf miner [5], and anthracnose [6], which pose severe threats to cucumber growth by impairing photosynthesis and nutrient absorption, ultimately reducing the yield. Therefore, the accurate detection and localization of pathological features on cucumber leaves during their growth cycle are essential for timely, precise, and quantitative pesticide application.

Currently, traditional methods for detecting pests and diseases on cucumber leaves can be broadly categorized into two approaches: conventional pathological methods and modern molecular biology techniques [7]. The conventional pathological methods involve experienced agricultural experts or farmers diagnosing potentially diseased cucumber plants by examining changes in their leaves, stems, and roots. Techniques, such as pathogen isolation and microscopic morphology analysis, are employed, but these methods are labor-intensive and prone to errors. Modern molecular biology techniques involve extracting DNA from diseased cucumber leaves or sending samples to a laboratory for pathogen identification using a polymerase chain reaction (PCR) and sequence analysis. While these methods improve the detection sensitivity and specificity, they require advanced equipment, technical expertise, and involve complex, time-consuming, and costly procedures.

In recent years, advancements in image processing and detection algorithms have led to the emergence of neural network-based methods for crop disease recognition. Huang et al. [8] proposed a knowledge-distillation-based plant disease detection method to improve model performance, achieving a mean average precision (mAP50) of 60.4% on the lightweight PlantDoc dataset, outperforming the existing approaches. Rangarajan et al. [9] used improved AlexNet and VGG16 models to train on tomato disease images, but their models lacked sufficient detection accuracy. Li Zimou et al. [10] introduced an Inception module into YOLOv3 to locate the leaf regions and combined it with a Faster R-CNN to detect pests and diseases on rose leaves. While this approach effectively reduced the impact of complex backgrounds, it involved a cumbersome process, and the YOLOv3 model was too large and slow for real-time detection. Wang Weixing et al. [11] proposed the YOLOv4-GCF model for detecting lychee pests and diseases. Despite its high recognition rate, the model suffered from large memory consumption and slow detection speed. Chen et al. [12] developed an improved Faster-RCNN-based cucumber leaf disease detection model using ResNet-50 as the feature extraction network and a Feature Pyramid Network (FPN) for multi-scale feature integration. This model achieved an mAP above 80%, but the processing speed was only 1.79 frames per second per image. Li et al. [13] (2023) proposed YOLOv9, which demonstrated outstanding performance in real-time object detection tasks by enhancing the feature extraction capabilities. Wang and Zhang [14] (2023) applied YOLOv9 to agricultural scenarios, showcasing its potential for pest and disease detection. Additionally, Chen et al. [15] (2024) introduced YOLOv10, which incorporated a Transformer architecture to further improve the model’s efficiency and accuracy.

While the above methods have their advantages, they also exhibit limitations, such as limited detection accuracy, large model sizes, high computational demands, and low frame rates. Moreover, in real-world cucumber cultivation scenarios, varying shapes and sizes of lesions, as well as widespread leaf occlusion, present significant challenges that existing methods fail to address effectively.

To address these issues, this study builds upon the YOLO model, focusing on the latest YOLOv8 network. Compared to its predecessors, YOLOv8 offers better detection performance, broad applicability, reduced computational requirements, and multiple model sizes for different applications, making it more suitable for agricultural and industrial use. A new SEDCN-YOLOv8 network model is proposed and compared against mainstream object detection algorithms, such as Faster-RCNN, SSD, YOLOv5, YOLOv6, and YOLOv7-tiny, to evaluate its performance and effectiveness.

## 2. Materials and Methods

### 2.1. Image Acquisition of Cucumber Leaf Pests and Diseases

This study focuses on two common and easily recognizable pests and diseases on cucumber leaves: target spot disease and leaf miner fly. Under the guidance of relevant professionals, image data were collected during two separate sessions at the Guangxi Academy of Agricultural Sciences and the Hunyuan Village cucumber plantation in Lingchuan County. The data collection took place in late October 2023 and in the spring of the following year. These periods were chosen because they coincided with the peak occurrence of pests and diseases in cucumbers, ensuring that the dataset reflected the typical seasonal manifestation of these issues.

Huawei smartphones were used as the imaging devices, with a camera resolution of 4096 × 3072 pixels. To build a cucumber leaf pest and disease dataset that reflects the complexity of natural backgrounds, images were captured during different times of the day (from afternoon to evening) under various natural lighting conditions, including sunlight, darkness, front light, back light, close up, long distance, and top-angle shots.

The resulting dataset contains clear images of healthy leaves, early-stage diseased leaves, mid-stage diseased leaves, and severely infected leaves, representing various stages of disease progression. It also includes other leaves and composite backgrounds, such as soil, weeds, other leaves, and canvas, reflecting the natural conditions of a plantation. As a result, the dataset incorporates diverse scenarios with varying light intensities, different leaf sizes, complex backgrounds, and overlapping leaves, ensuring both the diversity of the data and the model’s ability to generalize.

**Figure 1:**
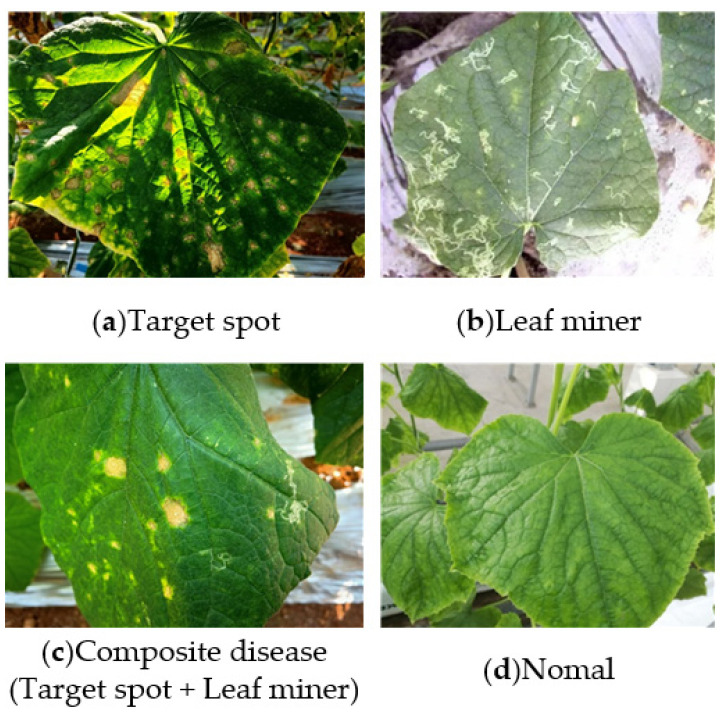
Cucumber leaf pests and diseases.

After filtering out images of poor quality and those unrelated to the diseases being studied, the final dataset comprises 1086 images. Each image contains between 2000 and 3000 sample instances. Furthermore, during data preprocessing, the model applies random cropping, rotation, scaling, and stitching operations, further enhancing the dataset’s diversity and ensuring the scale of the dataset. Figure 1 shows some of the leaf samples associated with the diseases being studied. As shown in Figure 1, the lesions on the cucumber leaves infected with target spot disease are mostly round or irregular in shape, with a yellow-brown color, resembling a target. In contrast, the lesions on the leaves infected by the leaf miner fly are irregular, curved, and white deformed lines.

### 2.2. Dataset Construction

**Figure 2:**
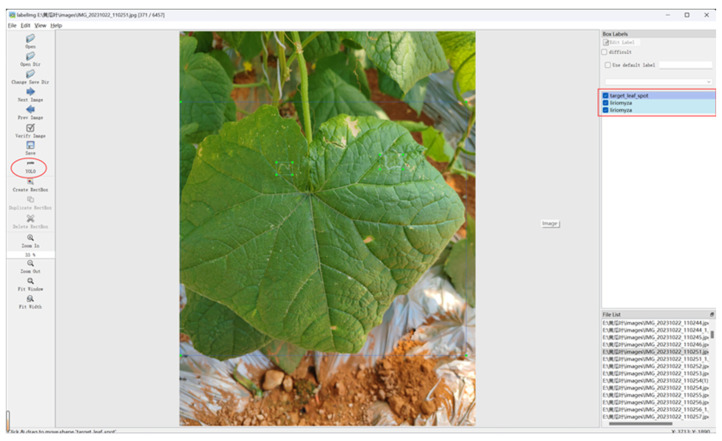
Images using LabelImg software.

As shown in Figure 2, LabelImg software was used to annotate the 1086 cucumber leaf disease and pest images. First, the save option was set to the YOLO format. Then, the disease-affected leaves and pest features were carefully outlined with bounding boxes, ensuring they perfectly matched the affected areas. After setting the corresponding labels, clicking save generated and stored the YOLO format labels in a .txt file. The content of the YOLO format labels in the .txt file is represented as [class ID, center coordinates x, center coordinates y, width w, height h], i.e., [n, x, y, w, h]. To effectively evaluate the model’s performance and generalization capability, the original dataset was randomly and evenly divided into training, validation, and test sets in an 8:1:1 ratio. This produced the final dataset for training and validation.

As shown in Table 1, the data samples are evenly distributed across the three subsets.

## 3. Algorithm Model and Evaluation Metrics

### 3.1. YOLOv8 Network Model

As a newer version of the single-stage object detection network, YOLOv8 introduces several enhancements compared to earlier YOLO versions. The YOLOv8 architecture is primarily composed of three main components: the Backbone (feature extraction network), the Neck (feature fusion network), and the Head (detection network).

The Backbone is responsible for feature extraction, incorporating a Bottleneck module, which is a variant of a residual block [16]. This module is primarily integrated into the C2f structure. The Bottleneck module is designed to improve network efficiency and representational capability while reducing the computational overhead. Its usage enables deep feature extraction and retains critical input feature information through residual connections, enhancing the network’s expressive power and optimizing the training process.

The Backbone also includes the SPPF module [17], introduced by Glenn Jocher (the author of YOLOv5), as an improvement over the SPP module [18]. SPPF is significantly faster than SPP. It performs pooling operations on the input feature maps at multiple scales, capturing multi-scale contextual information and fusing these features to provide richer representations for the subsequent network layers.

The Neck is primarily tasked with feature fusion, combining features from different levels of the Backbone to create a unified feature representation.

The Head employs a decoupled head design to independently handle classification and regression tasks. This approach allows each branch to specialize in its respective task, thereby improving the overall model accuracy. The detection head utilizes CIOU and DFL loss functions to process the bounding box losses, particularly enhancing performance at detecting small objects.

While the original YOLOv8 model has demonstrated strong performance on cucumber leaf pest and disease detection tasks, there remain areas for improvement. Specifically, challenges persist in handling multi-scale and deformed lesions, as well as occluded diseased leaves, which require further optimization.

### 3.2. Improved YOLOv8 Model

This paper proposes a new YOLOv8 network structure, named **SEDCN-YOLOv8**, incorporating three key improvements. The modifications to SEDCN-YOLOv8 are as follows:

(1) Integration of Deformable Convolution (DCNv2)
  The last CBS block in the Bottleneck module is replaced with a deformable convolution (DCNv2) [19] block, reconstructing it into a Bottleneck_DCNv2 block. Subsequently, the Bottleneck_DCNv2 block replaces the original Bottleneck block in the C2f module, forming the C2f_DCNv2 module. This improved C2f_DCNv2 module is then used to replace the C2f module located immediately before the SPPF module in the Backbone of the original YOLOv8 model.
(2) Enhanced Attention with SEAM
  The second CBS block in the original Detect module of the detection head is replaced with a Separated and Enhanced Attention Module (SEAM) [20], reconstructing it into a Detect_SEAM detection head. This newly constructed detection head replaces the original Detect module in the YOLOv8 model.
(3) Optimization with Focaler-SIOU Loss
  The original CIOU Loss [21] function used in the YOLOv8 model is replaced with the Focaler-SIOU Loss [22,23] function.

**Figure 3:**
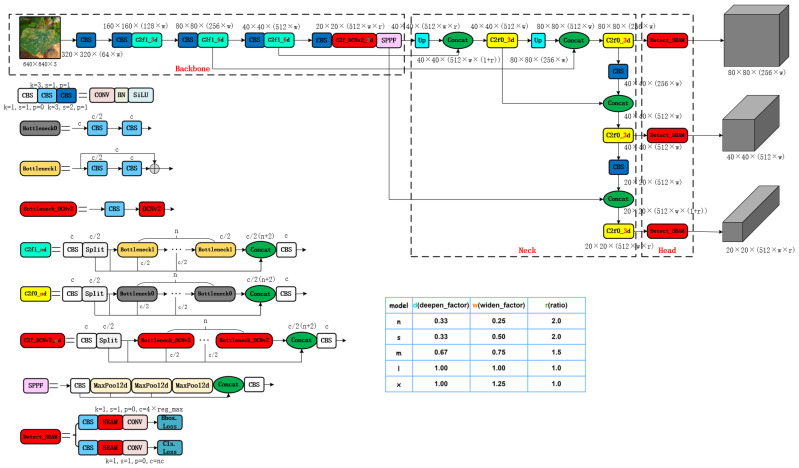
SEDCN-YOLOv8 Network Architecture Diagram. Note: The deep-red modules in the structural diagram represent the improved modules compared to those in the original YOLOv8. CONV stands for convolution; BN stands for batch normalization; siLU refers to the siLU activation function; Concat indicates feature fusion; Split denotes feature channel separation; UP refers to upsampling; “⊕” indicates feature channel addition; MaxPool2d represents max pooling; DCNv2 stands for deformable convolution; SEAM represents Separable Enhanced Attention Module; Bottleneckn, Bottleneck_DCNv2, C2fn_nd, C2f_DCNv2_nd, SPPF, and Detect_SEAM are composite modules made up of basic modules.

The network architecture of the SEDCN-YOLOv8 model is illustrated in Figure 3.

#### 3.2.1. Deformable Convolution DCNv2

The lesions and diseased leaves of cucumber plants are typically characterized by a variety of shapes and sizes, with significant variations in both their dimensions and forms. The standard convolutional kernels in the original YOLOv8 have fixed receptive field sizes and shapes, which limits their ability to effectively capture the features of disease spots and diseased leaves.

In the original YOLOv8 network model, the Bottleneck module serves as the core of the feature extraction network, and is responsible for multi-level feature fusion and abstraction. To enhance the model’s adaptability to complex shapes and deformations before performing high-level abstraction, we propose replacing the final CBS module within the Bottleneck of the previous C2f module in the SPPF of the feature extraction network with a deformable convolution (DCNv2). This modification ensures that the DCNv2 can refine the features that have been initially extracted, leading to improved detection accuracy.

**Figure 4:**
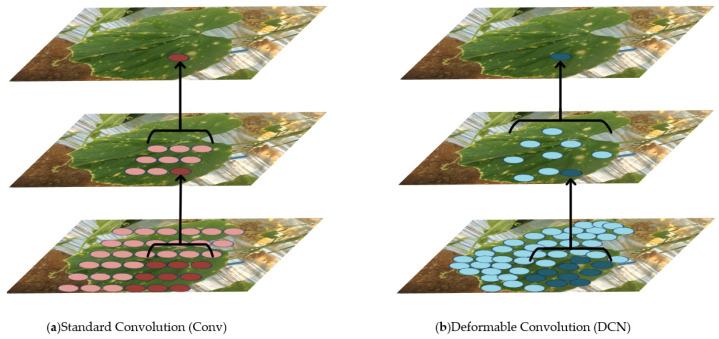
Visualization of DCNv2 effects.

As a result, during the feature extraction process, the receptive field of the convolution kernel will more closely align with the shape, size, and dimensions of the disease features and diseased leaves, thereby enhancing the detection precision. Moreover, this modification significantly improves the model’s ability to accurately locate and recognize deformable targets. Figure 4 effectively demonstrates the advantage of DCNv2 over standard convolution kernels, showing how DCNv2’s receptive field is much better suited to the size, shape, and dimensions of the detection targets.

The basic principle of deformable convolution is as follows:

For an input feature map of size W × H × C, a standard convolution operation first generates a 3D tensor containing the horizontal and vertical offsets for each pixel of the original feature map. The size of this tensor is W × H × 2N, where N represents the number of pixels in each dimension of the convolution kernel.

Each pixel in the resulting 3D tensor contains a 2N-dimensional vector, representing the horizontal and vertical offsets of the N pixels. These pixel offsets are then applied to the input feature map (with bilinear interpolation used during this process to ensure smooth fitting), producing a deformed input feature map.

This deformed feature map is then processed by a standard convolution operation, yielding the final output feature map, which has the same size as the input feature map (W × H × C).

**Figure 5:**
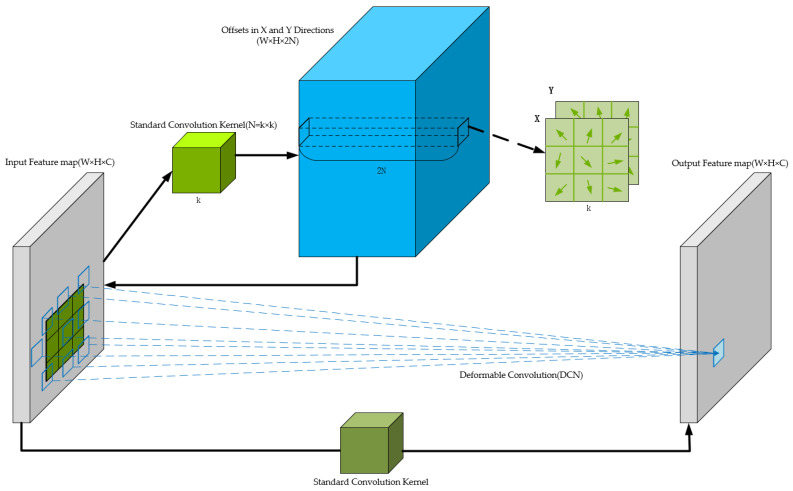
Structural diagram of DCNv2 principle.

A structural diagram of the deformable convolution principle is shown in Figure 5.

#### 3.2.2. Separated and Enhanced Attention Mechanism (SEAM)

In complex cucumber cultivation scenarios, leaf occlusion among diseased plants is a significant challenge. The initial YOLOv8 network model lacks the mechanisms to address such occlusion. As a result, when used to detect diseased leaves in environments with complex backgrounds, where they often appear in clusters or groups, the original YOLOv8 model exhibits noticeable limitations. To overcome this issue, the Separated and Enhanced Attention Mechanism (SEAM), which has shown strong performance in handling occlusion problems, is integrated into the original YOLOv8 network. The integration of SEAM was inspired by its use in YOLO-FaceV2 to address facial occlusion, and is adapted here to tackle the occlusion of diseased cucumber leaves. In the original YOLOv8 model, the Detect module is responsible for the final detection tasks, including classification and regression. The introduction of SEAM into the second CBS block of the Detect module enhances attention to key features before the final classification and regression stages. This improvement helps the model to better recognize and localize targets, especially those that are partially occluded. The original detection head, Detect, is replaced with a modified detection head, Detect_SEAM, incorporating the SEAM.

The SEAM network module also offers a lightweight effect. Its internal depthwise separable convolution decomposes a standard convolution operation into a depthwise convolution and pointwise convolution. This decomposition significantly reduces both the number of parameters and the computational cost of the model. For example, for a convolution layer with an input and output channel both equal to *c*, and a kernel size of *k*, the parameter count for a standard convolution is c * c * k * k, while for a depthwise separable convolution, it is reduced to c * k * k + c * c, which represents a considerable reduction. In the SEAM module, the use of depthwise separable convolutions significantly lowers both the parameter count and the computational load.

The SEAM network module compensates for the response loss from the occluded leaves by enhancing the response of unobstructed leaf regions, thereby improving the detection accuracy for occluded leaves. SEAM achieves this through a combination of depthwise separable convolutions and residual connections. Depthwise separable convolutions operate on individual channels, enabling a network to learn the importance of each channel while reducing the parameter count. However, it overlooks the relationships between channels. To address this limitation, outputs from different depthwise convolutions are combined via pointwise (1 × 1) convolutions. A two-layer fully connected network is then applied to merge the information from all the channels, strengthening the inter-channel relationships. By learning the connections between the occluded and non-occluded regions, the model compensates for the occlusion-induced losses.

**Figure 6:**
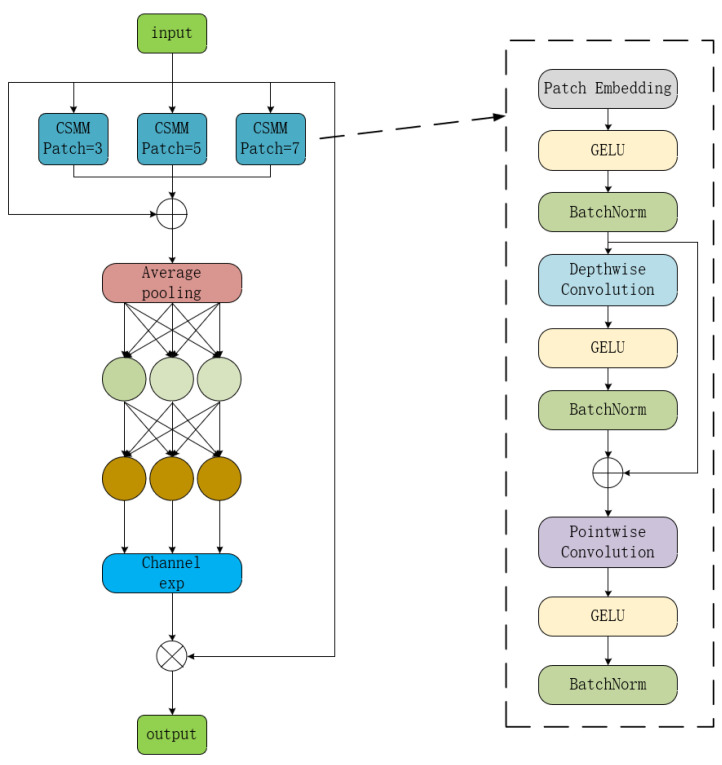
SEAM structure diagram.

The basic structure of the SEAM network module is shown in Figure 6. The module operates as follows: First, the input feature map passes through three CSMM modules, each with different patch parameters (patching refers to padding operations on the feature map). The outputs of these modules are added to the input feature map, followed by average pooling. The pooled feature map then passes through two fully connected layers twice to generate an adjusted feature map, which is multiplied with the input feature map to produce the final output.

#### 3.2.3. Focaler-SIOU Loss Function

Traditional loss functions in object detection (such as IOU, GIOU, DIOU, CIOU, and EIOU) rely on aggregated metrics for the bounding box regression, including the distance between the predicted and ground truth boxes, overlap area, and aspect ratio. However, the existing methods fail to account for the mismatch direction between the desired ground truth box and the predicted box. This limitation results in slower convergence and reduced efficiency, as the predicted box may “wander” during training, ultimately leading to a suboptimal model.

SIOU introduces a novel loss function that redefines the penalty metric by considering the angular vector between the expected regression direction and the actual movement. To address these challenges, this study incorporates the Focaler-SIOU as a replacement for the CIOU loss function used in the original YOLOv8 model.

**Figure 7:**
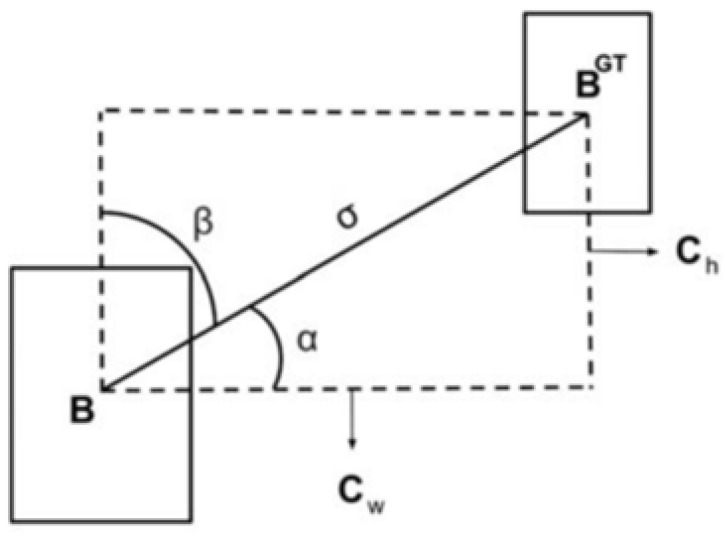
Angle loss calculation.

The relevant calculation formulas for SIOU are as follows:

Angle cost:

(1) $$\Lambda =1-2*{sin}^{2}(\alpha -\frac{\pi }{4})$$

where α is the angle shown in Figure 7.

Distance cost:

(2) $$\Delta =\sum_{t=x,y}\left(1-e^{-\gamma \rho t}\right)$$

where

(3) $$\rho_{x}={\left(\frac{b_{cx}^{gt}-b_{cx}}{C_{w}}\right)}^{2}$$

(4) $$\rho_{y}={\left(\frac{b_{cy}^{gt}-b_{cy}}{C_{h}}\right)}^{2}$$

(5) γ=2−Λ

**Figure 8:**
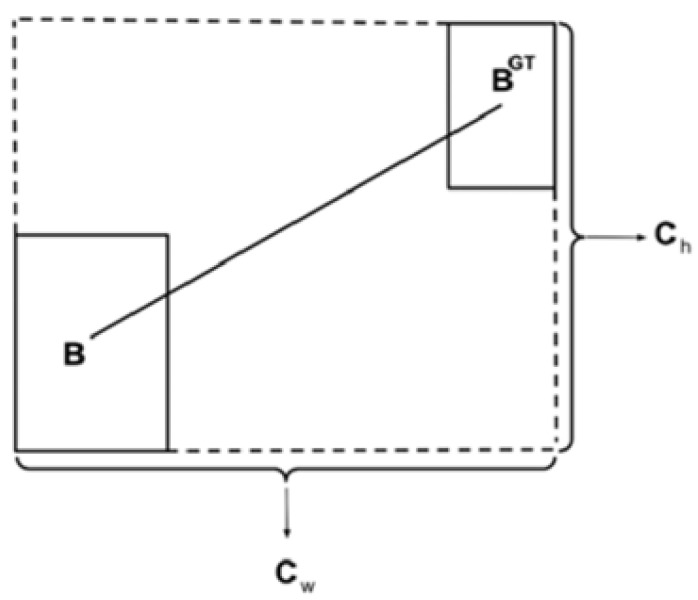
Distance loss calculation.

As shown in Figure 8, $b_{cx}^{gt}$ and $b_{cy}^{gt}$ represent the center coordinates of the ground truth box, $b_{cx}$ and $b_{cy}$ represent the center coordinates of the predicted box, and $c_{w}$ and $c_{h}$ represent the width and height of the minimum enclosing rectangle for the ground truth and predicted boxes.

Shape cost:

(6) $$\Omega =\sum_{t=w,h}{\left(1-e^{-w_{t}}\right)}^{\theta }$$

where

(7) $$w_{w}=\frac{\left|w-w^{gt}\right|}{max(w,w^{gt})}$$

(8) $$w_{h}=\frac{\left|h-h^{gt}\right|}{max(h,h^{gt})}$$

and *w, h*, $w^{gt},\;and\;h^{gt}$ represent the width and height of the predicted box and ground truth box, respectively. θ is an adjustable parameter used to control the emphasis placed on shape loss.

The final calculation formula for the SIOU loss function is

(9) $$L_{SIOU}=1-IOU+\frac{\Delta +\Omega }{2}$$

The Focaler-SIOU incorporates the concept of Focal Loss, addressing the imbalance of training samples in the bounding box (BBox) regression. Specifically, in a single image, the number of high-quality anchor boxes with small regression errors is significantly fewer than the low-quality samples with large errors. Low-quality samples can generate excessively large gradients that negatively impact the training process.

The Focal Loss mitigates this issue by separating the high-quality and low-quality anchor boxes from a gradient perspective, introducing an additional penalty term, ${IOU}^{\gamma }$, where γ is a parameter controlling the degree of suppression for outliers.

This Focal component differs from the traditional Focal Loss, which increases the loss for harder samples, aiming to mine difficult examples. In contrast, the penalty term in the Focaler-SIOU assigns a higher loss to samples with higher IOU values, effectively acting as a weighting mechanism. This approach emphasizes better regression targets, leading to improved regression precision.

Thus, the final calculation formula for the Focaler-SIOU is

(10) $$L_{Focal-SIOU}={IOU}^{\gamma }L_{SIOU}$$

### 3.3. Model Training and Evaluation Metrics

#### 3.3.1. Experimental Environment Configuration

The experimental environment parameters used in this study are shown in Table 2.

#### 3.3.2. Training Parameter Settings

To ensure the smooth execution of the experiment, it is essential to set the hyperparameters appropriately. The specific experimental parameter settings are as follows: the input image size (img_size) is set to 640 × 640, the batch size (batch_size) to 32, the number of worker threads (workers) to 2, the learning rate (lr) to 0.01, the pretrained weights (pretrained) to false, the early stopping patience (patience) to 100, and the number of training epochs (epoch) to 600. These settings are detailed in Table 3 below.

The batch size refers to the number of images fed into the network model during each training iteration. It is typically set as a power of two, depending on the available GPU memory.

Workers refers to the number of threads used for data loading, and it is set based on the system’s RAM capacity.

The learning rate is a crucial parameter in training. If it is set too low, the training process becomes very slow. On the other hand, setting it too high may speed up the training, allowing the model to converge more quickly. However, it also increases the risk of overshooting the optimal minimum, which could lead to poor model performance or even gradient explosion.

Given that the task in this study involves non-mainstream scene elements, the official pretrained weights are not suitable for this particular task, and thus, we do not use pretrained weights.

The early stopping parameter patience is typically set to 50 by default. However, to avoid missing the opportunity for improving the detection metrics, we choose a higher value. Specifically, if there is no improvement in the metrics within 100 training epochs, the model will stop training. This helps prevent overfitting while ensuring that we do not miss the optimal model metrics. Once the training metrics stop improving within the set patience range, training will be concluded early.

The epoch value is set to a sufficiently large number to ensure proper training convergence, while the patience parameter works alongside it to avoid overfitting. This combination ensures both stable convergence and the prevention of overfitting.

#### 3.3.3. Evaluation Metrics

To evaluate the model’s performance at detecting cucumber leaf pests and diseases under natural and complex backgrounds, this study employs common evaluation metrics used in deep learning [24]. The primary metrics include the following: precision (P, %), recall (R, %), mean average precision at IoU threshold 50 (mAP50, %), mean average precision across IoU thresholds from 50 to 95 (mAP50-95, %), floating-point operations (FLOPs), model size (MB), and frames per second (FPS).

Precision (*P*) represents the ratio of correctly predicted positive samples to all the predicted positive samples. The calculation formula is as follows:

(11) $$P=\frac{T_{P}}{T_{P}+F_{P}}\times 100\%$$

Recall (R) represents the ratio of correctly predicted positive samples to all the actual positive samples. The calculation formula is as follows:

(12) $$R=\frac{T_{P}}{T_{P}+F_{N}}\times 100\%$$

The mean average precision at 50% IoU (mAP50) is the mean value of the average precision (AP50). The AP50 is defined as the area under the precision–recall (P-R) curve when IoU = 0.5. The calculation formula is as follows:

(13) $${mAP}_{50}=\frac{\sum_{i=1}^{N}\int_{0}^{1}P(R)dR}{N}\times 100\%$$

The mean average precision at IoU thresholds from 0.5 to 0.95 (mAP50-95) is calculated by taking the mAP at each IoU threshold, incremented by 0.05, and then averaging the results. The mAP50 is considered a general metric for evaluating model performance, while the mAP50-95 serves as an absolute measure of performance.

In the formulas mentioned above, the variables are as follows:

$T_{P}$ represents the number of correctly detected samples with cucumber leaf diseases and pests in the dataset.

$F_{P}$ represents the number of samples incorrectly identified as diseased or pest-affected cucumber leaves.

$F_{N}$ represents the number of actual diseased or pest-affected cucumber leaves missed during detection.

N denotes the number of disease and pest categories. In this study, there are two categories for cucumber leaf diseases and pests, i.e., N = 2.

## 4. Results and Analysis

To analyze the effectiveness of the proposed improvements for enhancing the model’s performance, four sets of experiments were designed under identical training hyperparameter settings for all the models:

1. Comparison of performance with mainstream object detection models;
2. Ablation study on the proposed improvements;
3. Heatmap comparison experiment;
4. Image inference comparison experiment.

### 4.1. Comparison Experiment

In this experiment, we selected the more recent versions of the YOLO series models, YOLOv11n, YOLOv10n [25], and YOLOv9t [26], along with earlier YOLO object detection models closer to the v8 version, YOLOv7-tiny [27], YOLOv6n [28], and YOLOv5n. Additionally, other classic object detection models, such as SSD [29] and Faster-RCNN [30], were included for comparison.

**Figure 9:**
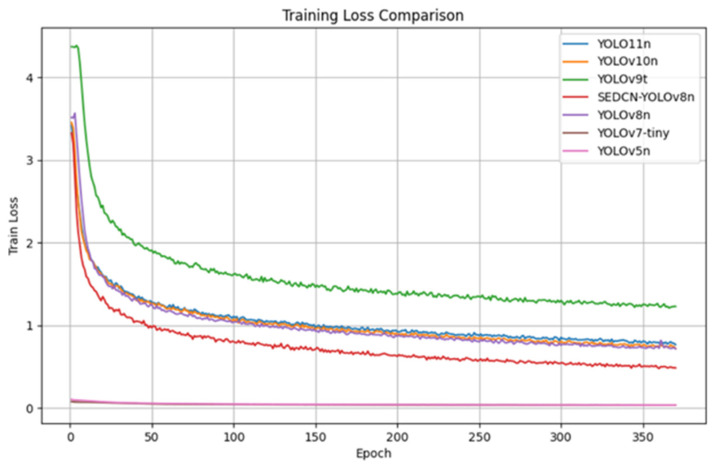
Comparison of loss curves.

**Figure 10:**
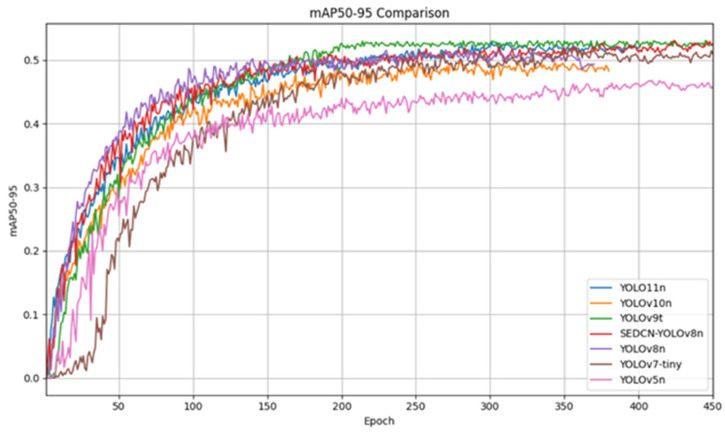
Comparison of mAP50-95 curves.

The training and testing were conducted on the same dataset with identical hyperparameter settings for up to 600 epochs to ensure model convergence. Figure 9 presents a comparison of the training loss curves for each model, while Table 4 summarizes the experimental results of the model comparisons. Figure 10 shows the comparison of the mAP (mean average precision) results for all the models.

From the comparison of the loss curves, it can be observed that all the models gradually converge, with the loss curve of SEDCN-YOLOv8n reaching a relatively low level. Due to the differing magnitudes of the loss curve metrics for YOLOv7-tiny and YOLOv5n compared to the other models, these two models start at a lower point on the curve from the beginning.

From the comparison experiment results, it is evident that the proposed network model, SEDCN-YOLOv8, achieves the highest mean average precision (mAP) of 53.1%, outperforming the other models. Specifically, it surpasses YOLO11n, YOLOv10n, YOLOv9t, YOLOv8n, YOLOv7-tiny, YOLOv6n, YOLOv5n, SSD, and Faster-RCNN by 0.4, 3.1, 0.3, 1.5, 1.1, 4.0, 6.0, 33.2, and 18.4 percentage points, respectively. This demonstrates that SEDCN-YOLOv8 delivers the best detection performance for cucumber leaf disease and pest detection tasks in complex natural environments.

In terms of the floating-point operations (FLOPs), SEDCN-YOLOv8 reduces the computations by 1.3, 3.8, 1.2, 6.1, 4.17, 266.71, and 394.81 GFLOPs compared to YOLOv10n, YOLOv9t, YOLOv8n, YOLOv7-tiny, YOLOv6n, SSD, and Faster-RCNN, respectively, while being only 0.6 and 2.8 GFLOPs higher than YOLO11n and YOLOv5n. Regarding the model size, SEDCN-YOLOv8 achieves reductions of 0.1, 0.3, 6.3, 3.35, 85.1, and 515 MB compared to YOLOv9t, YOLOv8n, YOLOv7-tiny, YOLOv6n, SSD, and Faster-RCNN, respectively, with only a slight increase of 0.5, 0.2, and 2.1 MB over YOLO11n, YOLOv10n, and YOLOv5n.

In terms of the inference speed, SEDCN-YOLOv8 demonstrates significant improvements, being 65.5, 149.4, 390.7, 255.0, 358.4, 395.27, and 383.32 fps faster than YOLOv10n, YOLOv9t, YOLOv7-tiny, YOLOv6n, YOLOv5n, SSD, and Faster-RCNN, respectively. It only lags behind YOLO11n and YOLOv8n by a marginal 21.1 and 35.2 fps.

These results clearly indicate that the SEDCN-YOLOv8 network model displays superior performance at detecting cucumber leaf diseases and pests under complex natural backgrounds. It effectively handles scenarios involving multi-scale, deformable lesions and overlapping diseased leaves. With 6.9 GFLOPs and a model size of only 6.0 MB, SEDCN-YOLOv8 ranks among the most efficient mainstream models. Moreover, its inference speed reaches 400 fps, meeting the requirements for real-time detection in industrial deployments.

### 4.2. Ablation Experiment

To evaluate the independent and combined effects of the improvements introduced in this study for cucumber leaf disease and pest detection, six sets of ablation experiments were designed for analysis and verification. Since the improved model does not significantly impact the detection speed—and the comparative experiment results have already demonstrated that the detection speed exceeds 400 fps—the FPS was excluded as an evaluation metric in the ablation study. Furthermore, since replacing the loss function has an independent impact on model performance, only the fifth and sixth groups focused on experiments related to the loss function. The final results of the ablation study are shown in Table 5.

#### 4.2.1. Analysis of Results of Ablation Experiments

1. Improvement from DCNv2

As shown by experiment 2, introducing a deformable convolution (DCNv2) into the backbone feature extraction network improves the mAP50 and mAP50-95 by 1.1 and 1.4 percentage points, respectively, compared to the baseline model in experiment 1.

This demonstrates that DCNv2 effectively addresses the detection challenges posed by the multi-scale and irregularly shaped lesions and leaves in cucumber leaf disease detection tasks.

2. Improvement from SEAM

Experiment 3 reveals that incorporating the Separable Enhanced Attention Module (SEAM) into the detection head results in mAP50 and mAP50-95 improvements of 1.1 and 0.8 percentage points, respectively, compared to the baseline model.

This indicates that the SEAM enhances detection in complex natural scenarios with highly occluded diseased cucumber leaves.

Notably, the inclusion of the SEAM reduces the computational cost (GFLOPS) by 1.1G and the model size by 0.4MB, attributable to the depthwise convolution [31] used in the CSMM module of the SEAM, which has fewer computations and parameters compared to the standard convolution.

3. Combined Effects of DCNv2 and SEAM

Experiment 4 shows that integrating both the DCNv2 and SEAM results in mAP50 and mAP50-95 improvements of 1.5 and 1.4 percentage points, respectively, confirming the positive synergy between these enhancements for addressing the baseline YOLOv8 model’s deficiencies.

4. Effectiveness of Focal-SIOU Loss

Experiment 5 demonstrates that replacing the original CIOU loss function with the Focal-SIOU improves the mAP50 and mAP50-95 by 1.2 and 1.6 percentage points, respectively.

This validates the Focal-SIOU’s effectiveness for improving regression accuracy for bounding box predictions.

5. Comprehensive Improvements with All Modifications

In experiment 6, the combined application of all three improvements (DCNv2, SEAM, and Focal-SIOU) yields a final detection performance of P = 79.2%, mAP50 = 75.2%, and mAP50-95 = 53.1%, which are increases of 7.2, 1.9, and 1.5 percentage points, respectively, compared to the baseline model.

Additionally, the computational cost (GFLOPS) and model size are reduced to 6.9G and 6.0MB, representing reductions of 1.2G and 0.3MB, respectively, compared to the baseline model.

#### 4.2.2. Visualizing Performance Differences

**Figure 11:**
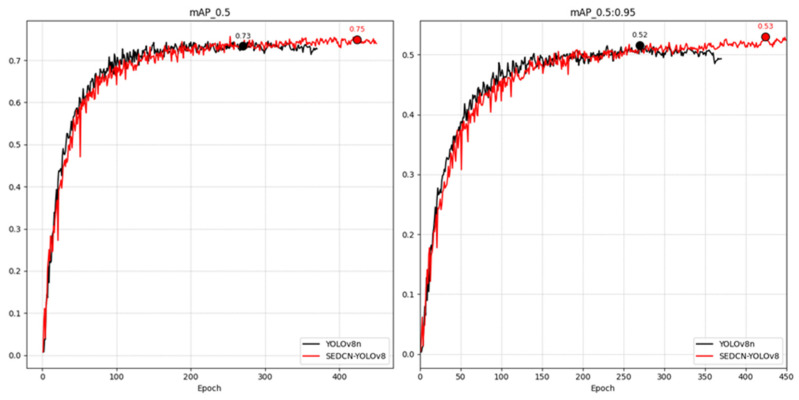
Comparison of model mAP trend curves.

To better illustrate the comprehensive performance differences between the SEDCN-YOLOv8 model and the YOLOv8n model, Figure 11 presents the trend curves for the mAP50 and mAP50-95.

From the graph, it is evident that under the 600-epoch training setting with an early stopping parameter of 100 epochs, both models converge early.

The peak values of mAP50 and mAP50-95 for the SEDCN-YOLOv8 model are higher than those for YOLOv8n, indicating the superior performance of the improved model on cucumber leaf disease detection tasks.

Therefore, based on the comprehensive analysis and validation of the ablation experiments, it can be concluded that the independent and combined effects of each improvement are positively effective.

### 4.3. Heatmap Comparison Experiment

To vividly demonstrate the effectiveness of the newly introduced module structures in the SEDCN-YOLOv8 network model, this experiment was designed. A representative image of cucumber leaf diseases and pests was selected, and heatmaps were generated to visualize the importance of the features the model focused on during inference and prediction.

The heatmaps were generated using the HiRes-CAM [32] technique, which analyzes the regions of interest a model focuses on for specific targets. It highlights the areas that contribute the most to the model’s decision-making process, visually identifying the regions of the detected image that have the greatest impact on predictions.

**Figure 12:**
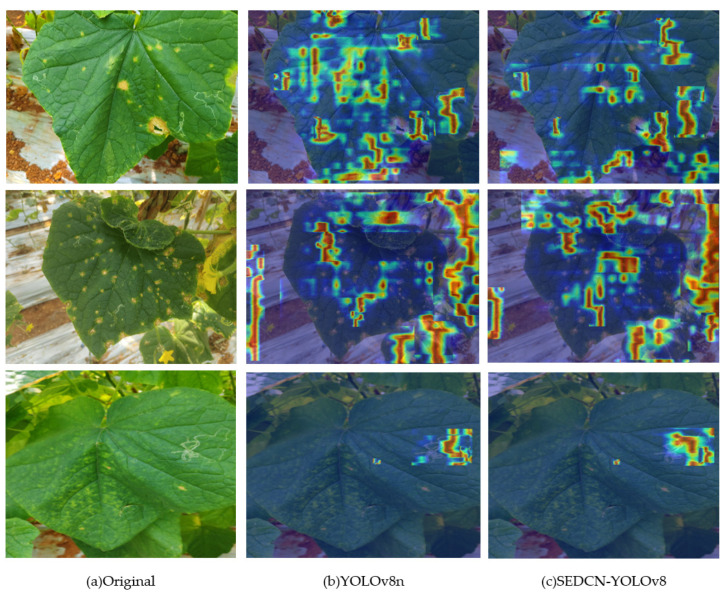
Heatmap comparison diagram.

Figure 12 illustrates the original image and the heatmap attention feature images generated by YOLOv8n and SEDCN-YOLOv8 for the same image.

From Figure 12, it can be observed that for the detection of leaf miner damage, SEDCN-YOLOv8 demonstrates significantly more shape- and size-aligned feature focus compared to YOLOv8n. This further indicates that the deformable convolution (DCNv2) effectively enhances the recognition of multi-scale, deformable lesions and leaves.

### 4.4. Image Inference Comparison Experiment

To visually demonstrate the superior detection performance of the SEDCN-YOLOv8 network model compared to the original YOLOv8, an image inference comparison experiment was designed. The selected images were classic examples from the test dataset, representing cucumber leaf diseases, such as target spot, leaf miner damage, healthy leaves, composite diseased leaves, and occluded leaves.

**Figure 13:**
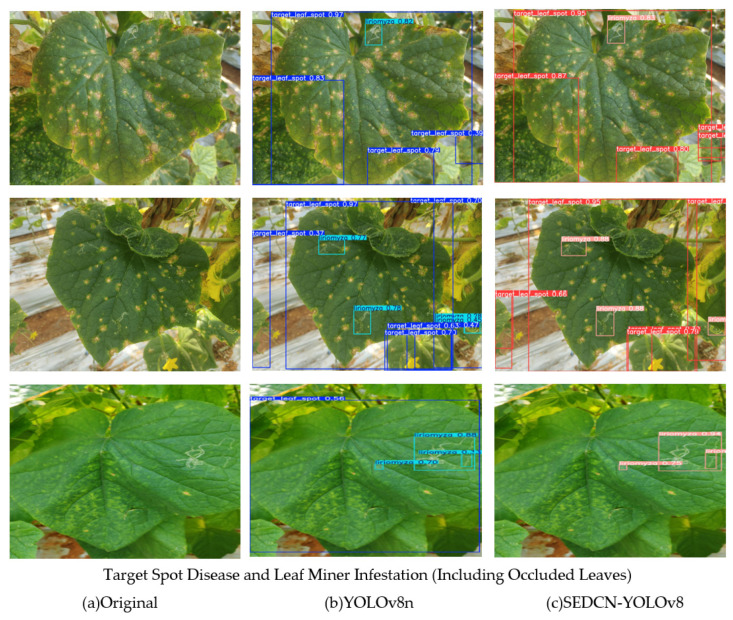
Comparison of image inference results.

Figure 13 below illustrates the visualized detection bounding boxes generated by the SEDCN-YOLOv8 and YOLOv8n models for these cucumber leaf disease images. The upper row shows the detection results for YOLOv8n, while the lower row presents the results for SEDCN-YOLOv8.

From the comparison, it can be observed that the upper-row images exhibit obvious false detections, whereas the lower-row images demonstrate bounding boxes that more closely match the true size and dimensions of the targets, especially for detecting occluded leaves. The results from the SEDCN-YOLOv8 model generally display higher accuracy. However, both models still exhibit some degree of missed detections.

## 5. Conclusions

To address the significant limitations of the existing models at detecting cucumber leaf diseases and pests in complex natural environments, this paper proposed an improved model based on YOLOv8, named SEDCN-YOLOv8, designed to overcome these challenges.

1. Key Improvements in SEDCN-YOLOv8

Backbone Enhancement: The C2f module preceding the SPPF module in YOLOv8’s Backbone was replaced with a C2f_DCNv2 module equipped with deformable convolution capabilities. This upgrade improved the detection accuracy and localization of irregular and large-scale lesions, as well as of diseased leaves.

Detection Head Update: The original detection head was replaced with a Detect_SEAM head, incorporating a Separable Enhanced Attention Module. This enhancement strengthened the response to both occluded and non-occluded surfaces, significantly improving the detection accuracy in cases where the diseased leaves were partially occluded.

Loss Function Optimization: the CIOU loss function was replaced with the Focal-SIOU, effectively improving the regression precision.

2. Performance Metrics

The final SEDCN-YOLOv8 model achieved an accuracy (P) of 79.2%, mAP50 of 75.2%, and mAP50-95 of 53.1%, marking improvements of 7.2%, 1.9%, and 1.5%, respectively, compared to the original YOLOv8 model.

The model’s computational complexity was reduced to 6.9 GFLOPS, and its size to 6.0 MB, representing reductions of 1.2 GFLOPS and 0.3 MB, respectively.

The detection speed reached 400 frames per second (f/s).

3. Conclusions

In summary, SEDCN-YOLOv8 demonstrates superior performance at detecting cucumber leaf diseases and pests in complex natural environments. Its compact size, low computational requirements, and high detection speed make it well-suited for industrial deployment in agricultural scenarios, providing technical support for the automation of cucumber disease and pest detection.

## Figures and Tables

**Table 1:** Cucumber leaf pest and disease dataset.

	Target Spot/ Instances	Leaf Miner/ Instances	Total Instances /Instances	Number of Images/Count
Training Set	2061	2349	4410	867
Validation Set	256	301	557	118
Test Set	244	298	542	101

**Table 2:** Parameters of the experimental environment.

Name	Version
Operating System	Windows11
Graphics Processing Unit (GPU)	NVIDIA GeForce RTX 4060
Central Processing Unit (CPU)	Intel Core i7-13700H/2.40 GHz
RAM	16 G
VRAM	8 G
Deep Learning Framework	Pytorch-2.0.1
Python	Python-3.8.17
CUDA	CUDA-11.8
CuDNN	CuDNN-8.6.0

**Table 3:** Experimental hyperparameter settings.

Parameter	Value
img_size	640 × 640
batch_size	32
workers	2
lr	0.01
pretrained	false
patience	100
epoch	600

A brief explanation of the above parameters is as follows:

**Table 4:** Results of mainstream model comparison experiments.

Model	mAP50-95 (%)	FLOPs (GFLOPS)	Model Size (MB)	Frame Rate (fps)
SEDCN-YOLOv8	53.1	6.9	6.0	400.0
YOLO11n	52.7	6.3	5.5	421.1
YOLOv10n	50.0	8.2	5.8	334.5
YOLOv9t	52.8	10.7	6.1	250.6
YOLOv8n	51.6	8.1	6.3	435.2
YOLOv7-tiny	52.0	13.0	12.3	9.3
YOLOv6n	49.1	11.07	9.35	145
YOLOv5n	47.1	4.1	3.9	41.6
SSD	19.9	273.61	91.1	4.73
Faster-RCNN	34.7	401.71	521	16.68

**Table 5:** Ablation experiment results.

Scheme 2	DCNv2	SEAM	Focal -SIOU	P /%	R /%	mAP50 /%	mAP50-95/%	GFLOPS/G	Model Size/MB
1				72.0	69.0	73.3	51.6	8.1	6.3
2	√			78.1	63.9	74.4	53.0	8.0	6.3
3		√		71.8	70.3	74.4	52.4	7.0	5.9
4	√	√		75.9	67.6	74.8	53.0	6.9	6.0
5			√	78.2	68.2	74.5	53.2	8.1	6.3
6	√	√	√	79.2	64.9	75.2	53.1	6.9	6.0

## Data Availability

The dataset used in this study was collected by the author team under the guidance of experts at a cucumber plantation in Hongyuan Village, Lingchuan County, and at the Guangxi Academy of Agricultural Sciences.
